# The effect of dilution with glucose and prolonged injection time on dexamethasone-induced perineal irritation – A randomized controlled trial

**DOI:** 10.1515/med-2022-0556

**Published:** 2022-09-24

**Authors:** Yonghai Zhang, Hong Liang, Liwei Huang, Junwei Zheng, Yi Chen, Bin Li, Fan Yang, Jingfang Yu, Hanxiang Ma

**Affiliations:** Department of Anesthesiology, General Hospital of Ningxia Medical University, Yinchuan, Ningxia, China; Operating Room, General Hospital of Ningxia Medical University, Yinchuan, Ningxia, China; Department of Anesthesiology, Renmin Hospital, Hubei University of Medicine, Shiyan, Hubei, China; Department of Anesthesiology, Ningxia Medical University, Yinchuan, Ningxia, China; Department of Anesthesiology, General Hospital of Ningxia Medical University, 804 ShengLi Street, Xingqing Area, Yinchuan, Ningxia, China

**Keywords:** dexamethasone, dilution, pain, pruritus

## Abstract

Dexamethasone can be used to prevent nausea and vomiting after surgery, but there is concern that it may induced perineal irritation. The aim of this study was to investigate the attenuation effect of dilution and slow injection on dexamethasone-induced perineal irritation. In this prospective, randomized, double-blind study, a total of 400 patients were enrolled and allocated into four groups: Group I, receiving 2 mL dexamethasone (5 mg/mL), Group II, receiving 5 mL dexamethasone (2 mg/mL), Group III, receiving 10 mL dexamethasone (1 mg/mL), and Group IV receiving 20 mL dexamethasone (0.5 mg/mL). Dexamethasone was diluted with 5% glucose. The injection time of dexamethasone was less than 2 s in Group I, while it was 30 s in Groups II, III, and IV. The incidence, onset, duration, and severity of perineal irritation were recorded. The incidence of dexamethasone-induced perineal irritation was 49, 33, 17, and 15% in Groups I, II, III, and IV, respectively. Group IV had less severity than Group I in mild and moderate perineal irritation (*P* < 0.008). The onset and duration of perineal irritation of Groups II, III, and IV were significantly improved compared to Group I (*P* < 0.001). Dexamethasone-induced perineal irritation can be alleviated by dilution of dexamethasone to 0.5 mg/mL with 5% glucose combined with prolonged injection time of 30 s.

## 1 Introduction

Dexamethasone is widely used to patients with general anesthesia [1], which aim to prevent and treat postoperative nausea and vomiting (PONV) [2,3]. However, there is a concern that intravenous bolus dose of dexamethasone may cause severe perineal irritation, with rapid and short-lived perineal pruritus, pain, and burning as its major clinical manifestations, particularly in vulnerable populations such as female patients [4–9]. Our previous study reported that the rate of dexamethasone-induced perineal irritation was 47.5%, and most of them experienced moderate pain [10]. In addition, the rate was described as high as 100% in female patients [7]. Therefore, anesthesiologists need to recognize this drug’s adverse effect and take therapeutic intervention immediately in high-risk patients. Pretreatment with dezocine [10] or fentanyl [8] or lidocaine [11] or dilution and slow injection [12–15] was found to have effect to reduce dexamethasone-induced perineal irritation. In clinical settings, an effective method that can not only guarantee the efficacy of the dexamethasone but also keep simple and convenient is necessarily needed. According to the drug instruction, dexamethasone can only be diluted by glucose. Besides, we also found that patients rarely experience perineal irritation when dexamethasone was slowly instilled after being diluted with glucose in clinic. Therefore, we hypothesized that dilution with glucose and prolonged injection time of dexamethasone might alleviate dexamethasone-induced irritation before the induction of general anesthesia.

## 2 Methods

The study, performed at the General Hospital of Ningxia Medical University from July 2021 to September 2021, was approved by the Institutional Ethics Committee of the General Hospital of Ningxia Medical University (2020-531), and written informed consent was obtained from all subjects participating in the trial. The trial was registered prior to patient enrollment at clinicaltrials.gov (https://clinicaltrials.gov/, registration number: NCT04950049).

### 2.1 Subjects

We conducted a single-center, prospective, randomized-controlled, double-blind study trial comparing the effect of dilution with glucose and prolonged injection time on dexamethasone-induced perineal irritation before general anesthesia induction. The study was conducted in accordance with applicable CONSORT guidelines. Participants were randomized in blocks of five stratified by sex in a 4-arm, parallel design with a 1:1:1:1 allocation ratio. A total number of 400 patients (male:female = 1:1) with American Society of Anesthesiologists (ASA) physical status I–III, an age between 18 and 65 years, a body mass index (BMI) of 18–24 kg/ m^2^, scheduled for elective surgery under general anesthesia were enrolled in this study. Exclusive criteria included contraindication or allergy to steroid, drug or alcohol abuse, diagnosed with paresthesia or mental diseases, communication disorders, endocrine disorder, pregnancy, or nursing. According to the gender of the patient, Dr Ma prepared randomization list, concealed group assignments in consecutively numbered, sealed, opaque envelopes together with detailed information of the study drug preparation. Thereafter, he was no longer involved in the study. A nurse selected randomization list based on the patient’s gender, and prepared study medication outside the operating room. The randomization was not disclosed to any of the personnel who observed the study parameters or the patient throughout the study.

No premedication drugs were given, and all the patients were instructed to fast for at least 8 h before the induction of anesthesia. After entering the operation room, all the patients were established with an 18-G cannula in the dorsum vein of the right hand, and Ringer Lactate was infused at a rate of 4–6 mL/min. Electrocardiogram, non-invasive blood pressure, and pulse oximeter were obtained.

Patients were randomly assigned into four groups and all received 10 mg dexamethasone (product batch no.: 2106213, Sinopharm Ronshyn Pharmaceutical Co. Ltd, Jiaozuo, China): Group I receiving 2 mL dexamethasone (5 mg/mL), the injection time of dexamethasone was less than 2 s; Group II receiving 5 mL dexamethasone (2 mg/mL) diluted with 5% glucose, the injection time of dexamethasone was 30 s; Group III receiving 10 mL dexamethasone (1 mg/mL) diluted with 5% glucose, the injection time of dexamethasone was 30 s; and Group IV receiving 20 mL dexamethasone (0.5 mg/mL) diluted with 5% glucose, the injection time of dexamethasone was 30 s.

### 2.2 Monitoring indicators

An anesthesiologist who was unaware of study medication observed and asked immediately if patients felt pain or pruritus during the first 5-min period after the injection of dexamethasone. The primary outcome was the incidence of perineal irritation. The secondary outcomes were onset, duration, and severity (based on Visual Analogue Score [VAS]) of perineal irritation if the patients felt discomfortable.

The day before surgery, all the patients were explained about the use of VAS scale to grade the severity of perineal irritation on the scale marked with 0–10. We defined the range of perineal pruritus including vagina, vulva, anus, scrotum, and penis. The severity of perineal irritation was evaluated based on the VAS as none (VAS 0), mild (VAS 1–3), moderate (VAS 4–6), or severe (VAS 7–10).

### 2.3 Statistical analysis

Statistical analysis was performed by Statistical Product for Social Sciences (SPSS) software (IBM SPSS Statistics, Version 25, IBM Germany, Ehningen, Germany). We tested continuous date for normal distribution using Q–Q plots and Shapiro–Wilk test and equality of variance using variance ratio test and Levene test. The age, weight, onset, and duration of pain are presented as mean with SD and were analyzed using one-way analysis of variance and least significant difference multiple comparisons. The comparison of incidence, severity, and ASA physical status were carried out using Chi-square test with Bonferroni correction. *P* < 0.05 was considered statistically significant.

Sample size estimation was performed using NCSSPASS software (version 11.0.7, update time January 22, 2013). It was calculated based on our previous study that the incidence of perineal irritation was 48%. We considered a 10% reduction with the gradual decrease of drug concentration as clinically important. This was based on a contingency table (chi-square tests) using 95% power and a 0.05 two-sided type I error probability. A sample size of 329 in all was required, to account for potential withdrawal or incomplete data, 400 patients were planned in all.

## 3 Results

**Figure 1:**
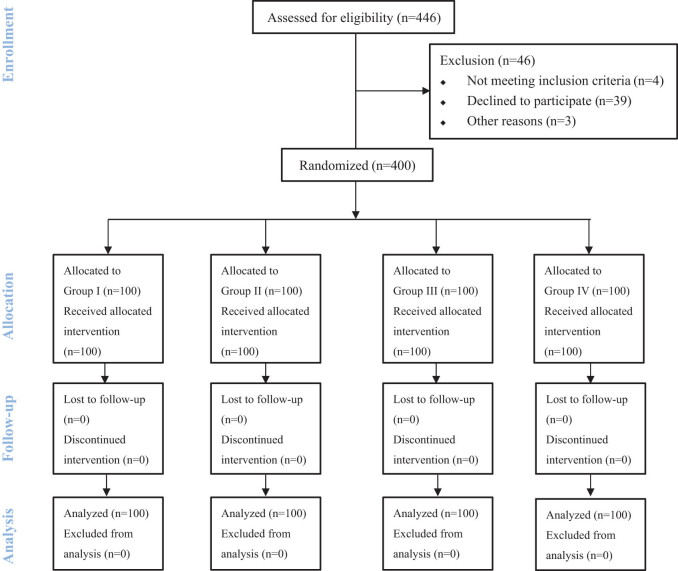
Enrollment and randomization of the patients.

The CONSORT study flow diagram is presented in Figure 1. Enrollment took place between July 15, 2021 and September 28, 2021. All patients completed this study as required by the protocol. There was no statistically significant difference between the four groups with regard to age, BMI, and ASA physical status, as shown in Table 1.

**Table 1:** Patient characteristics

Variables	Group I	Group II	Group III	Group IV	*P*-value
	(*n* = 100)	(*n* = 100)	(*n* = 100)	(*n* = 100)	
Age (year)	44.6 ± 12.0	47.3 ± 11.4	47.0 ± 12.1	45.4 ± 12.7	0.334 (>0.05)
BMI (kg/m²)	21.4 ± 1.8	21.5 ± 1.7	21.2 ± 1.7	21.4 ± 1.6	0.493 (>0.05)
ASA (I/II/III)	24/65/11	18/67/15	22/66/12	19/64/17	0.830 (>0.05)

*P* > 0.05, non-significant. Data shown are mean (SD) or absolute numbers. ASA, American Society of Anesthesiologists; BMI, body mass index.

For primary outcome, the incidence of dexamethasone-induced perineal irritation was 49, 33, 17, and 15% in Groups I, II, III, and IV, respectively. Groups III and IV had a lower incidence than Group I (*P* < 0.008). Groups IV had a lower incidence than Group II (*P* < 0.008). There was no significant difference in the incidence between Groups I and II, between Groups II and III, and between Groups III and IV (*P* > 0.008). The incidence of perineal irritation was significantly higher in females than in males in Groups I and II (*P* < 0.001), as shown in Table 2.

**Table 2:** The incidence of dexamethasone-induced perineal irritation

		Group I	Group II	Group III	Group IV	*P*-value
Incidence (%)	Total (*n* = 100)	49 (49)	33 (33)	17 (17)^*^	15 (15)^*†^	0.0001 (<0.01)
	Male (*n* = 50)	13 (26)	8 (16)	6 (12)	5 (10)	0.130 (>0.05)
	Female (*n* = 50)	36 (72)^‡^	25 (50)^‡^	11 (22)^*†^	10 (20)^*†^	0.0001 (<0.01)

*P* > 0.05, non-significant; *P* < 0.01, highly significant. Data shown are absolute numbers (%). Compared with Group I, ^*^a significance level of *P*＝0.008 was used (0.05/6 Bonferroni adjustment). Compared with Group II, ^†^a significance level of *P*＝0.008 was used (0.05/6 Bonferroni adjustment). Compared with male, ^‡^ *P* < 0.001, statistical significance was defined as *P* < 0.05.

Secondary outcomes are shown in Tables 3 and 4. In the severity of dexamethasone-induced perineal irritation, Groups III and IV had less severity than Group I in mild perineal irritation (*P* < 0.008), and Groups IV had less severity than Group I in moderate perineal irritation (*P* < 0.008). In the onset and duration of dexamethasone-induced perineal irritation, Group I had shorter time than Groups II, III, and IV in onset (*P* < 0.001), and Group I had longer time than Groups II, III, and IV in duration (*P* < 0.001).

**Table 3:** The severity of dexamethasone-induced perineal irritation

		Group I	Group II	Group III	Group IV	*P*-value
Severity	None	51 (51)	67 (67)	83 (83)^*^	85 (85)^*†^	0.0001 (<0.01)
	Mild	29 (29)	18 (18)	11 (11)^*^	10 (10)^*^	
	Moderate	18 (18)	14 (14)	6 (6)	5 (5)^*^	
	Severe	2 (2)	1 (1)	0 (0)	0 (0)	

*P* < 0.01, highly significant. Data shown are absolute numbers (%). Compared with Group I, ^*^a significance level of *P*＝0.008 was used (0.05/6 Bonferroni adjustment). Compared with Group II, ^†^a significance level of *P*＝0.008 was used (0.05/6 Bonferroni adjustment).

**Table 4:** The onset and duration of dexamethasone-induced perineal irritation

Variables	Group I	Group II	Group III	Group IV	*P*-value
	(*n* = 100)	(*n* = 100)	(*n* = 100)	(*n* = 100)	
Onset (s)	28.3 ± 7.8	43.6 ± 9.1^*^	44.6 ± 9.8^*^	41.9 ± 10.6^*^	0.0001 (<0.01)
Duration (s)	61.8 ± 11.8	48.6 ± 13.2^*^	46.9 ± 11.8^*^	45.1 ± 12.7^*^	0.0001 (<0.01)

*P* < 0.01, highly significant. Data shown are mean (SD). Compared with Group I, ^*^ *P* < 0.01. Statistical significance was defined as *P* < 0.05.

## 4 Discussion

Intravenous dexamethasone-induced perineal irritation has been commonly observed during induction of anesthesia [5–10]. Besides, it has been described when it is used as an anti-inflammatory agent [14], during treatment of severe head injury [16] and as an antiemetic drug in chemotherapy [13]. Most previous studies indicated that the incidence of dexamethasone-induced perineal irritation ranges from 30 to 100%, and this discrepancy can be explained by differences in injection dose and time [7–9]. In Dylla study [4], its incidence was reported to be as low as 2.7%, probably mainly because patients were only counted when they voluntarily reported these events, which could lead to reporting bias. The mechanism of dexamethasone-induced perineal irritation remains unclear but may relate to phosphate ester since perineal irritation has been described with hydrocortisone 21-phosphate sodium [17] and prednisolone phosphate [18]. In addition, the short duration of perineal irritation might represent the time required for the compound to be hydrolyzed to phosphate ions and dexamethasone [19]. Wang et al. [11] speculated that dexamethasone may mediate the pathogenesis of pruritus through activation of the sodium channels in peripheral unmyelinated C-fiber polymodal afferents within the superficial layers of skin and mucous membrane. However, the etiologies and pathophysiology of the side effects remain unknown and further research is necessarily required.

The results of our study showed that when dexamethasone was diluted ten times with 5% dextrose and given 15 times slower, peritoneal irritation findings were seen 34% less compared to concentrated and rapid administration. In addition, we found that females had higher incidence than males, which was consistent with the previous studies [8–12]. The mechanism by which dilution with glucose and prolonged injection time reduces the incidence of perineal irritation remains unclear. We propose as a possible explanation for this phenomenon that dilution and prolonged injection time of dexamethasone may result in a lower concentration of phosphate ester. From the pharmacokinetic point of view, prolonged drug injection may affect the peak plasma concentration, with a longer injection time resulting in a smaller peak concentration, which may not reach threshold level of pathogenicity. The phenomenon of females having higher incidence than males maybe attributed to the threshold levels that were lower in females than in males.

Several measures have been applied to reduce the incidence of dexamethasone-induced perineal irritation. Rewari et al. [8] reported that pretreatment with fentanyl (1 µg/kg) can alleviate the rate from 71 to 25% and the severity of dexamethasone-induced perineal irritation. Gu et al. [12] found that dilution with normal saline to 0.5 mg/mL combined with prolonged injection time can eliminate the occurrence of perineal irritation. However, there is a concern that using the normal saline to dilute dexamethasone may destroy the structure and efficacy of the drug because dexamethasone can only be diluted by glucose according to the drug instruction. This also explains why it can completely eliminate perineal irritation, which is also inconsistent with our results. Another method of alleviating the perineal irritation was to inject dexamethasone after induction of anesthesia, but it was found [20] that the prophylactic intravenous administration of dexamethasone immediately before the induction, rather than at the end of anesthesia, was more effective in preventing PONV. Pretreatments with 1 or 1.5 mg/kg lidocaine [11] or 0.1 mg/kg dezocine [10] also can suppress the incidence and severity of dexamethasone-induced perineal irritation, but these two drugs were limited in terms of their clinical application. In addition, some early reports simply indicated that this adverse effect can be diminished or even abolished by infusion of a diluted solution of dexamethasone over a period of several minutes [13–15]. Our study showed that dilution of dexamethasone to 0.5–1 mg/mL with small dose of glucose combined with prolonged injection time of 30 s can effectively reduce the incidence, duration of perineal irritation, and frequency of mild and moderate perineal irritation, which can be used easily and inexpensively and almost unrestricted.

There are many factors associated with dexamethasone-induced perineal irritation, such as gender, drug dosage, concentration, and intravenous injection rate. Therefore, we chose a 10 mg dose in our study, the usually used clinical dose in operation. We also controlled the injection time of dexamethasone less than 2 s. As the incidence of dexamethasone-induced perineal irritation was higher among females than males, we controlled the sex-ratio in each group as 1:1.

There were also some limitations for our study. First, our sample size was limited, just dilute the drug concentration to a minimum of 0.5 mg/mL and prolong the injection time for 30 s, and so we may not find the optimal scheme to reduce the incidence of perineal irritation. Second, we only considered the impact of gender on the results, ignoring other factors such as age and weight.

## 5 Conclusion

In summary, dexamethasone administered intravenously appears to cause significant perineal irritation, dilution of dexamethasone to 0.5 mg/mL with 5% glucose combined with prolonged injection time of 30 s is an effective, inexpensive, and feasible method to reduce the incidence, duration, and severity of dexamethasone-induced perineal symptoms.
